# Utilization of health facilities and maternal malaria prevention strategies by pregnant women in Kajiado County, a highland pastoral area of Kenya with low malaria transmission

**DOI:** 10.5281/zenodo.10758234

**Published:** 2017-06-09

**Authors:** Jonathan C. Ngala, Erick K. Serem, Francis M. Gwama

**Affiliations:** 1 Department of Medical Laboratory Sciences, Kenyatta University, P.O. Box 43844-00100, Nairobi, Kenya; 2 Department of Public Health, Pwani University, P.O. Box 195-80108, Kilifi, Kenya; 3 Department of Nursing, Umma University, P.O. Box 713-01100, Kajiado, Kenya

## Abstract

**Background:**

During pregnancy, malaria poses a great health risk to both mother and foetus. In Kenya, to prevent and control infections, mothers receive intermittent preventive treatment during pregnancy (IPTp) and are provided with a bednet (ITN). Uptake of these control strategies, however, is not optimal. In Kajiado County, for instance, only ITNs are given to pregnant women, without IPTp. We assessed utilisation of health facilities and WHO-recommended maternal malaria control strategies in Kajiado County.

**Materials and methods:**

A total of eleven health facilities were recruited, in which 6899 pregnant women were divided in three groups. Group 1 were women attending a clinic and used ITNs, group 2 did not attend a clinic but used ITNs and group 3, which did neither. 86% Of deliveries were assessed; 84% of these in clinics and 16% at home. Throughout pregnancy, data on abortion and premature births were collected. Upon delivery, data on stillbirths, birth weight and neonatal mortality was noted. Mother’s cord and placental blood was examined for malaria parasites and parasitaemia using microscopy; haemoglobin levels were determined.

**Results:**

86% Of the women visited a health facility, 97% used an ITN. Only 3% went without visits or bednet usage. Although the number of cases was low, attending a clinic and using a bednet increased maternal Hb and reduced maternal mortality. Use of nets decreased maternal malaria cases and mortality due to malaria whilst maternal Hb increased. Across the study groups, infant outcomes improved, with fewer abortions, premature births, still births, neonatal mortality and an increase in mean body weight at birth.

**Conclusion:**

Women should be sensitised to visit clinics and use ITNs for better maternal and new-born health outcomes.

## 1 Introduction

Maternal malaria is a major public health problem, with substantial risks for both the mother and foetus. In subtropical areas with moderate to high transmission of *Plas-modium falciparum*, the World Health Organization (WHO) recommends a package of interventions for controlling malaria and its effects during pregnancy, which includes the promotion and use of insecticide-treated bed-nets (ITNs), the administration of intermittent preventive treatment with sulfadoxine-pyrimethamine (IPTp-SP), and appropriate case management through prompt and effective treatment of malaria [1]. During the last few years, WHO has observed a slowing of efforts to scale-up IPTp-SP in a number of African countries, including Kenya [2]. Although there may be several reasons for this, an important factor is confusion amongst health workers about sulfadoxine-pyrimethamine administration for intermittent preventive treatment in pregnancy. In a recent WHO evidence review [3], a meta-analysis of seven trials that evaluated IPTp-SP was undertaken. It showed that three or more doses of IPTp-SP were associated with higher mean birth weight and fewer low birth weight (LBW) births than two doses of IPTp-SP. The estimated relative risk reduction for LBW was 20% (95% CI 6-31%). This effect was consistent across a wide range of SP resistance levels. The 3+ dose group also had less placental malaria. There were no differences in serious adverse events between the two groups [4]. Based on this evidence review, in October 2012, WHO updated the recommendations on IPTp-SP as outlined below, and urged national health authorities to disseminate this update widely in order to ensure its correct application. IPTp-SP is an integral part of WHO’s three-pronged approach to the prevention and treatment of malaria in pregnancy, which also includes the use of ITNs and prompt and effective case management. There are, however, challenges facing the uptake and utilisation of these strategies [3]. This may be even worse in pastoralist communities where inhabitants keep on shifting from one place to another. Interventions adopted by clinics in Kajiado County do not issue sulfadoxine-pyrimethamine prophylactic treatment as recommended by WHO because of low malaria transmission. However, urbanisation and climate change has been found to alter *Anopheles* population and malaria transmission dynamics [5]. Kajiado is growing rapidly, with massive construction of modern housing infrastructure with gutters and water reservoirs. This therefore, formed the basis for a survey to determine malaria parasite infection levels, parasitaemia, and use of clinical facilities and ITNs in relation to maternal and infant health outcomes.

## 2 Materials and methods

### 2.1 Study site and population

The study was conducted in Kajiado County for a period of twelve months, from July 2015 to June 2016. The County is located at 2.0981˚S, 36.7820˚E with a population of 687,312 in an area of 21,293 km^2^ [6]. It is elevated at 1582 m above sea level bordering Nairobi and extending to the Tanzanian border in the South. This area is semi -arid with seasonally flowing rivers. Two rainy seasons are experienced; the long rains between April and June and short rains between October and December. Annual mean rainfall is low, between 500 and 1250 mm with livestock agriculture as the main economic activity. Native inhabitants are mainly pastoralists. Temperatures range between 14˚C to 34˚C. In general, the area has low malaria indices but with the county becoming more developed in addition to climatic changes, some cases have been observed. A total of 11 health facilities were selected to represent the whole County. Among these were one County referral hospital, three Sub County hospitals and seven health centres offering maternity services.

### 2.2 Study design, recruitment and exclu sion criteria

Pregnant women within Kajiado County were informed of the study and a written consent form signed. The sample size for this study was based on similar previous studies in Kenya [7] and Uganda [8]. Three groups were identified from this sample of 6,899 pregnant women. The first group (n=5,910) consisted of women attending a clinic and using malaria prevention strategies. The second group (n=794) comprised pregnant women not attending clinic but using ITNs. The third group (n=195) comprised pregnant women not attending a clinic and not using an ITN. It is important to note that this latter group of women declined to adopt any interventions despite sensitisation efforts. Blood parameters (parasitaemia and haemoglobin level) were also assessed. Similarly, information on women who were pregnant but not attending clinics was obtained from the local administration (area chiefs). Assessment tests and interviews were done with this group. Upon consent, these mothers were followed up to delivery to assess maternal and infant health outcomes. Pregnant women who attended an antenatal clinic past their 26^th^ week of pregnancy, those with history of adverse drug reactions, severely diseased with cases of anaemia (Hb<7.0g/dl), hepatitis, jaundice, tuberculosis or HIV were excluded from the study. However, these individuals were put on appropriate medication.

### 2.3 Utilization of ITNs

Women with pregnancies <26 weeks were interviewed on their malaria prevention methods and issued with ITNs during their first antenatal visit. Those not using any preventive measures were issued with an ITN and instructed on how to use it. Similarly, those not attending a clinic and consented to use ITNs were visited, issued with one and taught how to use it. Blind follow up visits were done to all groups to assess ITN usage.

### 2.4 Maternal malaria and parasitaemia

On delivery, blood samples were collected from the umbilical cord and placenta. Blood slides were stained with Giemsa and examined for malaria parasites. At least 200 oil immersion visual fields at a magnification of x1000 were examined on both thick and thin smears. The degree of parasitaemia was determined using both thin and thick smears. Total number of red cells and parasitized red cells were tabulated separately. The number of infected red cells in 1000 erythrocytes was noted and converted into percentages. If occasional parasites were seen when scanning the smear, but none identified during the process of counting 300-500 red blood cells, a percentage value of less than 1% of erythrocytes parasitized was assigned. Thick films were also prepared since they yield higher sensitivity than thin smears; with a detection level of 1050 trophozoites/μl thick smears are therefore better for estimating parasite concentrations. Infected erythrocytes were counted in relation to a predetermined number of white blood cells (WBC) and an average of 8000/μl taken as standard. 200 Leucocytes were counted in 100 fields (0.25μl of blood). All parasite species and forms including sexual and asexual forms were recorded. If >10 parasites were counted, then the following formula was applied: (# of Parasites/ # of WBCs counted) x 8000 = # of parasites/ μl. Or if 200 leukocytes were counted: # of parasites counted x 40 = # of parasites/μl. If the number of parasites counted was <9, then 500 WBCs were counted and the formula used was: # of parasites counted x 16 = # of parasites/μl.

### 2.5 Maternal anaemia

Anaemia was estimated by determining the concentration of haemoglobin in blood samples. For accuracy, Haemoglobin (Hb) concentration was measured using a Hemo-Cue photometer (HemoCue Corp., Angelholm, Sweden) [9]. Levels of Hb were classified into three categories: low <11g/dl, normal 12-18g/dl, high>18g/dl.

### 2.6 Infant health outcomes

Infant health outcomes were assessed using the following parameters: a) number of abortions, b) premature births, c) still births, d) birth weights and e) neonatal deaths. Abortion was classified as ‘birth’ at <6 months, premature birth at 6-9 months, low birth weight as <2,5 kg and neonatal deaths at <28 days. Gestation age at birth was estimated using Ballard external criteria [10,11] for all babies delivered at the health units. For home deliveries, babies were visited and weighed at home. Blood films were prepared from both the mother (placenta and cord) and baby (heel phlebotomy) for examination of parasitaemia. A follow up on both babies born in the health unit and at home was done up to 28 days to assess any early and late neonatal mortality and/or maternal deaths. Birth weights were determined using a digital scale with accuracy to the nearest 10 g. Data on potent relationships on maternal malaria/ infant outcomes and clinic utilisation/ITN usage were analysed using descriptive generalised regression models in SPSS. Relationships between two means were compared using t-tests.

## 3 Results

### 3.1 Use of maternal malaria prevention strategies

A total of 6899 pregnant women from various health units within the county were included as shown in Table 1. The number of deliveries assessed was less than that recruited because of migration, abortions, fatal premature births and maternal death cases that occurred during the study period. In general, more women (5910; 85.7%) accepted to use the clinic facilities, with 794 (11.5%) accepting only to use ITNs but not attend clinics. Only 195 (2.8%) declined to use both clinic attendance and ITNs. More pregnant women were using ITNs (M=305, SD=107) than those not using (M=161, SD= 7.8), t_32_=4.7, p<0.001. Both groups issued with ITNs were adhering to their proper use, F_1,20_=2.52, p<0.05.

**Table 1.** Women grouped by location and interventions adopted (group 1: clinic visit and ITN use; group 2: ITN use only; group 3: no intervention). Adherence to ITN use is also indicated.

Site	Group 1 (n)	Clinic attended	Deliveries assessed (%)	Adherence (%)	Group 2 (n)	Deliveries assessed (%)	Adherence (%)	Group 3 (n)	Deliveries assessed (%)	Total
1.Kajiado	1000	Referral	950(95)	948(95)	50	45(90)	43(86)	7	5(71)	1057
2.Kitengela	720	Sub County	660(92)	645(90)	55	50(91)	46(84)	10	10(100)	785
3.Loitoktok	700	Sub County	610(87)	593(85)	80	74(93)	70(88)	18	16(89)	798
4.Ngong	800	Sub County	730(91)	724(91)	60	56(93)	54(90)	15	14(93)	875
5.Rongai	400	Health centre	300(75)	297(74)	84	80(95)	76(90)	22	20(91)	506
6.Isinya	420	Health centre	340(81)	334(80)	65	62(95)	60(92)	20	18(90)	505
7.Bisil	370	Health centre	260(70)	252(68)	90	88(98)	85(94)	20	20(100)	480
8.Mashuru	390	Health centre	300(77)	255(65)	68	65(96)	55(81)	25	25(100)	483
9.Masimba	360	Health centre	250(69)	214(59)	78	75(96)	54(69)	34	34(100)	472
10.Rombo	380	Health centre	300(79)	264(69)	74	70(95)	56(76)	10	10(100)	464
11.Kimana	370	Health centre	270(73)	210(57)	90	85(94)	66(73)	14	14(100)	474
Sub total	5910		4970 (84)	4736 (80)	794	750 (94)	665 (84)	195	188 (96)	6899

### 3.2 Maternal blood parameters

Blood parameters measured at the time of delivery are shown in Table 2. Before and after the interventions, the number of *P. falciparum* cases were 2, 3 and 5 (before) and 2, 1, and 7 (after), for groups 1-3, respectively. Mean levels of Hb increased across all groups following the interventions. The effect of attending a clinic was analysed by comparing maternal blood parameters between group 1 and 2. There was a significant advantage of protection against *P. falciparum* infection by attending maternal clinic, t21=0.557, p<0.001. This was similar for the other parameters (parasitaemia, Hb and maternal mortality) (p<0.001). A higher number of *P. falciparum* and maternal mortality cases were reported in group 3 (7 and 6 respectively) with the lowest level of Hb recorded in that group (11.4g/dl). These numbers were significantly different when compared with those attending clinics and using malaria prevention strategies (p<0.001). Overall, low parasite infection levels, parasitaemia and mortalities were noted in Kajiado County.

**Table 2.** *P. falciparum* positive cases, Hb levels, and maternal mortality before and after the interventions (group 1: clinic visit and ITN use; group 2: ITN use only; group 3: no intervention).

	*P. falciparum* positive		Haemoglobin (g/dl) Before		Maternal mortality
	Before	After	Before	After	
Group 1	2	2	11	17	0
Group 2	3	1	9	14.8	3
Group 3	5	7	10	11.4	6

### 3.3 Infant health outcomes

In all sentinels, abortions and premature births were low with 1 and 2 cases respectively reported in group 3 (Table 3). Still births were at 0.08% in the whole County for the study period with 2 cases reported each in group 2 and 3. The mean birth weight was normal at 3.5 kg (SD 1.25) with highest birth weight at 4.8 kg in group 1. Neonatal mortality was at 0.22% of all deliveries assessed. Same numbers of neonatal mortality (6 cases) were reported in groups 2 and 3. Infant malaria was generally lower (<1%) across all groups.

**Table 3.** Infant health outcomes.

	Abortions	Premature births	Still births	Mean birth weight (kg)	Neonatal mortality
Group 1	0	0	1	4.8	1
Group 2	0	0	2	3.4	6
Group 3	1	2	2	2.3	6

## 4 Discussion

Pregnant women in Kajiado County embraced maternal prevention interventions, with a major preference for ITNs. A major challenge encountered was migration of candidates due to their pastoralist lifestyle and marriages for those who were pregnant in their maternal homes. Those not embracing the use of ITNs (n=195) cited myths like having bad dreams and lack of enough air when sleeping under nets (unpublished data). It was also observed that health facilities in this County do not administer SP as recommended by WHO with the argument that malaria transmission is very low. Most women living in urban centres and the towns Kajiado, Kitengela, Loitoktok, Rongai, Ngong and Isinya used and adhered to ITNs more than their counterparts in rural areas. This was attributed to the cosmopolitan nature of the sites and increase in awareness (through media, notably television) of the importance of ITNs and clinic attendance. In rural set ups, most ITNs were passed on to children by the women instead of using these themselves. In urban set ups, those not adhering were found to be using ITNs as poultry housing. Similar misuse of ITNs has been reported elsewhere in Africa [12].

Although numbers were very low in all three groups, the number of malaria infections among pregnant women using ITNs was lower than for those not using nets, indicating the importance of using nets [8]. Parasitaemia levels remained low throughout the study period, which is attributed to the high altitude of between 1500 to 1700 m above sea level, where transmission intensity is generally low [13]. Those neither attending a clinic nor using ITNs showed more cases at the end of the study. This may be due to sharing of common households (different sleeping rooms within same house/ different apartments within the same compound) with individuals from malaria endemic areas, mainly from coastal or western Kenya. Besides maternal malaria management, clinic attendance and ITNs are very important to pregnant women as they prevent mortalities due to pregnancy complications or other pathogenic infections. Similar information on the importance of ITNs was reported in a study from Uganda [8]. In the present study, women that attended clinics had the highest levels of Hb. This is likely because they got frequent health assessment and information on iron-based nutritional diet from the clinics. Similarly, those not attending clinics had a lower average level of Hb and this can be attributed to the common diet for inhabitants of this region. Being pastoralists, their diet comprise mainly of animal products thus boosting blood iron content.

The use of ITNs and attending clinic are important component for health infant outcomes. In this study, they appeared to reduce abortions, premature births, still births and neonatal mortality. Mean body weight for newborns increased. In other studies [8,14], similar advantages were reported although the latter reported ITNs protection in early infancy is not a risk factor for mortality in high malaria transmission areas. Higher numbers of abortions, still births and neonatal mortalities reported in those not attending clinic could be due to poor nutritional diets, heavy continuous labour, use of low effective traditional methods of midwifery and use of traditional medication as opposed to hospital services. Common infections worsening the situation were pneumonia and other respiratory syndromes due to, at times, a cold and dusty environment (unpublished data).

## 5 Conclusions

Use of ITNs is the widely accepted mode of malaria control and therefore should be encouraged, especially in the rural areas [15]. IPTp and ITNs should be used within urban and more populated areas because travellers can introduce malaria parasites in these places from high-transmission areas. More so, the climatic changes resulting in higher average temperatures and humidity may favour vector populations in these areas [5,16]. Urbanisation of most centres in the County has prompted construction of modern houses with water reservoirs and drainage channels. This may encourage breeding of vectors if not properly managed. Women should be encouraged to attend clinics for consistent health assessment, diagnosis and medications. In addition, pregnant women should not be subjected to heavy continuous labour as it is the case at present; husbands should assist or look for assistance from house helps or neighbours (unpublished report). This will promote most pregnancy to terms and reduce maternal mortality, abortions, still births and neonatal mortality.
